# Skin-Compatible Biobased Beauty Masks Prepared by Extrusion

**DOI:** 10.3390/jfb11020023

**Published:** 2020-04-06

**Authors:** Maria-Beatrice Coltelli, Luca Panariello, Pierfrancesco Morganti, Serena Danti, Adone Baroni, Andrea Lazzeri, Alessandra Fusco, Giovanna Donnarumma

**Affiliations:** 1 Consorzio Interuniversitario Nazionale per la Scienza e Tecnologia dei Materiali (INSTM), 50121 Florence, Italy; luca.panariello@ing.unipi.it (L.P.); adone.baroni@unicampania.it (A.B.); andrea.lazzeri@unipi.it (A.L.); alessandra.fusco@unicampania.it (A.F.); 2 Department of Civil and Industrial Engineering, University of Pisa, 56122 Pisa, Italy; serena.danti@unipi.it; 3 Academy of History of Health Care Art, 00193 Rome, Italy; morgantipf@gmail.com; 4 Dermatology Department, China Medical University, Shenyang 110001, China; 5 Department of Experimental Medicine, University of Campania “Luigi Vanvitelli”, 80138 Naples, Italy

**Keywords:** starch, poly(hydroxyalkanoate), biopolyesters, beauty masks, releasing, skin compatible

## Abstract

In the cosmetic sector, natural and sustainable products with a high compatibility with skin, thus conjugating wellness with a green-oriented consumerism, are required by the market. Poly(hydroxyalkanoate) (PHA)/starch blends represent a promising alternative to prepare flexible films as support for innovative beauty masks, wearable after wetting and releasing starch and other selected molecules. Nevertheless, preparing these films by extrusion is difficult due to the high viscosity of the polymer melt at the temperature suitable for processing starch. The preparation of blends including poly(butylene succinate-co-adipate) (PBSA) or poly(butylene adipate-co-terephthalate) (PBAT) was investigated as a strategy to better modulate melt viscosity in view of a possible industrial production of beauty mask films. The release properties of films in water, connected to their morphology, was also investigated by extraction trials, infrared spectroscopy and stereo and electron microscopy. Then, the biocompatibility with cells was assessed by considering both mesenchymal stromal cells and keratinocytes. All the results were discussed considering the morphology of the films. This study evidenced the possibility of modulating thanks to the selection of composition and the materials processing of the properties necessary for producing films with tailored properties and processability for beauty masks.

## 1. Introduction

Over the past two decades, declining fertility and mortality rates have resulted in an increased aged population [1]. This phenomenon has created room for innovation, leading to a robust demand for anti-aging products in order to prevent wrinkles, age-spots, dry skin, uneven skin tone and even hair weakening. By 2050, in fact, the worldwide population over 60 years of age is expected to reach 2.09 billion: the life expectancy for women has been predicted to rise from 82.8 years in 2005 to 86.3 in 2050, while for men this increase is expected to be from 78.4 to 83.6 years. As a consequence, new demographic trends, technologies, and consumer insights are impacting the cosmetic sector that has shown a general resistance to macroeconomic events such as recession [2]. Thus, today the global beauty industry reached a value of $532 billion, with the largest market represented by the US (with about 20% share), followed by China (13%) and Japan (8%). With a compound annual growth rate between 5% and 7%, the beauty market is estimated to reach $800 billion by 2025, remaining impervious to the ups and down of the global economy [2,3].

However, while 70% of women aged more than 40 years old want to see more beauty products targeting anti-aging issues, more specific men’s personal care products are also emerging. Moreover, consumers are increasingly demanding about the real composition of the products they are buying, and they wish to have more information regarding the use of the different cosmetics and beauty products that work both internally and topically, being less interested in the usual advertising and marketing announcements. Thus, ingredient sourcing and effectiveness have become a major concern for the cosmetic industry together with the use of recyclable and refillable packaging materials necessary to reduce their carbon footprint, especially if obtained by industrial and agro-food waste.

In the cosmetic sector, the consumers’ naturally oriented demand is towards the use of biobased active molecules and materials; this so called clean and natural beauty, therefore, is not only about what is in consumers’ products, but also about how products are produced and packaged. In conclusion, in moving the raw materials from waste and/or land to the lab, biotechnologies are increasingly impacting the production of future beauty ingredients as well as the final products. Biobased materials to make innovative beauty masks go in this direction because such renewable raw materials seem able to protect the skin from pollution and modulate its microbiota [4].

For all these reasons, the production of more and more commercial products made by biobased and biodegradable polymers [5] (especially those products having a brief life-cycle) can be no longer delayed.

Beauty masks are currently mainly produced by using wet, nonwoven tissues, often prepared with fossil-based fibers. After their use, these products and their packaging materials are not selectively collected, contributing to increase the nondifferentiated part of the urban waste, often incinerated or landfilled.

Biobased and biodegradable polymeric materials, with the capacity of adhering to skin after wetting and releasing active molecules that are skin-friendly and easily compostable after usage, represent highly eco-sustainable options and at the same time meet consumers’ expectations. They can be commercialized at the dry state, avoiding the use of preservatives [5,6,7]. Moreover, they can effectively release starch [8] together with the functional molecules previously added by several techniques to the starch or to the obtained film, for instance as coating. 

Among the biobased polymers already available on the market, poly(hydroxyalkanoates) (PHAs), a family of polymers obtained from bacteria [9], are suitable for this application because of their very high biocompatibility [10,11], lower greenhouse gas emissions [12] and both soil and marine compostability [13,14]. These properties make the PHA-based materials very promising to be used in applications where environmental concern and biocompatibility are both fundamental. On the other hand, starch is the major carbohydrate reserve in higher plants [15] and is also a very abundant biopolymer still much used in nonfood applications (e.g., glues, gums, thickener, soothing for skin).

Skin-compatible PHA/starch films including calcium carbonate were prepared in a previous paper [16], where a PHA elastomer (EM 5400F EM 5400F, obtained from Shenzhen Ecomann Biotechnology Co.), a specific calcium carbonate and an easy methodology to pregelatinize starch were investigated and selected for preparing films by compression molding. These films resulted as promising to make beauty masks being flexible enough to follow the skin curvature, being sticky when applied on wet skin and having a fast release kinetic in water. However, their preparation was based on compression molding of biopolymeric powder with calcium carbonate as additive. This methodology is different from the ones currently used in industry to prepare bioplastic flat films characterized by a high productivity [17,18,19] and therefore can limit the real feasibility of the process.

Moreover, the preliminary extrusion of the blend consisting of PHA, starch and calcium carbonate showed a high value of torque in agreement with a high viscosity of the melt. Therefore, the processing should be made easier to allow the use of these biobased films in industrial applications.

Poly(hydroxybutyrate) (PHB), the most investigated biopolyester of the PHA family, has a limited stability at the high temperatures required for the melting processes. It, in fact, undergoing thermal degradation affects its physical and mechanical properties, making the industrial process difficult [20]. The very low resistance to thermal degradation seems to be the most serious problem related to its processing. The main reaction involves chain scission, which results in a rapid decrease in molecular weight [21]. Copolymers of 3-hydrobutyrate with other hydroxyacids present a lower melting point and an increased processing window. However, the critical molecular weight of these polymers is high [22] and, when processed, it is necessary to control the temperature, keeping it slightly above the melting point to avoid the polymer degradation. Therefore, plasticization is generally adopted as the methodology to control the processing temperature [9].

The blending of PHA, especially PHB with starch, has been extensively investigated [23,24]. Godbole et al. [25] discussed the compatibility of PHB with starch to achieve the improved properties and reduce cost. The results revealed that films had a single glass transition temperature (Tg) for all proportions of PHB/starch blends. However, the necessity of increasing the compatibility of PHB and PHA with starch is evidenced in other papers, and several methodologies including the chemical modification of polymers are proposed [26,27].

The blending with copolyesters having a low viscosity in the processing temperature window of PHA seems to be a good strategy to enhance processability. Elastomeric commercial copolyesters are the most promising, while copolymers based on poly(butylenesuccinate) with adipic acid (PBSA) are commercially available. They are currently partially renewable, but they will be fully renewable in the near future [28], being biodegradable in composting plant and soil [29]. Thus, blends of PBS or PBSA with PHA were investigated for improving the compatibility [30], and their promising application in flexible packaging films was recently demonstrated [31].

Blends of PHB and PBS filled with starch were recently investigated by Zhang et al. [32]. It was evidenced that starch nanoparticles localized on the PHB/PBS phase interfaces improve phase adhesion, while those dispersed in the continuous PHB phase are able to prevent the coalescence of PBS droplets during the melt mixing, thus remarkably decreasing the droplet domain size. Although these results were achieved for specific nanocomposites, they suggest that ternary PHA/PBSA/starch blends can show an affordable compatibility. On the other hand, some authors have shown that blends of PBS with starch showed a good adhesion at the interface [33], with an improved processability [34] that enabled these blends to be used in food packaging.

Another biopolyester that can be blended with PHAs is poly(butylene adipate-co-terephthalate) (PBAT). Currently, it is obtained only partially from renewable sources, but in a short time it will be fully renewable [28], and it is biodegradable in a composting plant [35]. Consequently, it will be suitable for preparing bioplastic films, incorporating starch with mechanical properties suitable for food packaging applications [36,37].

Regarding the blending of PHA and PBAT, Larsson et al. [38] evidenced that PBAT can be a very efficient additive to improve the processability of PHA. Matos Costa et al. [39] studied the thermal behavior of the blends and evidenced that the crystallization of PBAT is very fast during cooling, whereas that of PHB is slow. Lin et al. [40] reported that these blends can be used in electrospinning to obtain antibacterial hydrophobic nanofibrous membranes. Zarrinbakhsh et al. [41] investigated the compatibilization of PHB/PBAT blends by considering the effect of a compatibilizer, polymeric methylene diphenyl diisocyanate (PMDI), and corn oil as lubricant for preparing composites, including a waste of bioethanol production. The change in melt processing force suggested the occurrence of chemical reactions during the process time. The glass transition peaks pertaining to the PBAT and PHBV matrix shifted slightly towards each other, suggesting the occurrence of crosslinking at the PBAT–PHBV interface due to the reactivity of PMDI. Belyamani et al. [42] investigated composites based on PHA/PBAT reinforced with trisilanolisobutyl polyhedral oligomeric silsesquioxanes (POSS) and calcium phosphate glass (CaP-g) under simulated physiological and human body temperature conditions. Biodegradation studies regarding PHB/PBAT blends [43] demonstrated that the amount of PBAT in the blend can impact the degradation rate, with formation of porous structure based on PBAT, but the material remains as substantially biodegradable as PBAT is.

The biocompatibility of materials for cosmetic use with the skin is a very important aspect in their production and marketing and mainly concerns the mechanisms of the innate immune response; the biological mediators of innate immunity are cytokines, which are multifunctional molecules implicated in various biological activities and endowed with pro- and anti-inflammatory activities. The best-studied members of this group are proinflammatory cytokines IL-1, tumor necrosis factor α (TNF-α), IL-6, chemokine IL-8 and anti-inflammatory cytokine transforming growth factor β (TGF-β). IL-1 promotes coagulation, increases the expression of adhesion molecules, causes the release of chemokines that recruit other leukocytes to the site of inflammation, and stimulates the growth and differentiation of B lymphocytes and of the many effector cell response [44,45]; The tumor necrosis factor α (TNF-α) is an essential mediator in inflammation [46]. The production of TNF-α and its release in the site of the inflammation involves a localized vascular endothelial activation, the release of NO and vasodilation with increased vascular permeability [44]; IL-6 is involved in synthesis of fibrinogen, which contributes to the inflammatory acute phase response [47], and IL-8 is involved in chemotaxis of basophils and has a role in angiogenesis [48]. Finally, The TGF-β or transformation and growth factor is part of the family of anti-inflammatory cytokines and is considered as the most powerful, able to negatively modulate almost all the inflammatory responses [49].

Coltelli et al. [16] described the use of a commercial elastomeric poly(hydroxyalkanoate) and starch for obtaining compression-molded bioplastic films with the necessary resistance in wet conditions, skin compatibility and capacity for a fast release of polysaccharides. Starting from these results, the preparation of blends by extrusion including poly(butylene succinate-co-adipate) (PBSA) or poly(butylene adipate-co-terephthalate) (PBAT) was successfully exploited in the present paper to better modulate melt viscosity to obtain films by extrusion. Their behavior and morphologies upon release in water were correlated using spectroscopic and microscopic evidence to understand the different releasing mechanisms. Consequentially, the biocompatibility of prepared films with cells was assessed by considering keratinocytes, and the mechanisms of the innate immune response were also investigated. The results were discussed considering the effect of extrusion and blending on the morphology of the films, their releasing capacity, their compatibility with cells and their immunomodulatory behavior. The objective is preparing a substrate suitable for adding specific functional molecules or complexes (in the starch phase or as coating) that can be released during the application of the mask onto skin.

## 2. Materials and Methods

### 2.1. Materials

PHA (Ecomann EM F5400 F) was supplied as pellet from Shenzhen Ecomann Biotechnology Co., Ltd., Shandong, China.

PBAT (Ecoflex C1200) was purchased as pellet from BASF. It is a statistical, aromatic–aliphatic copolymer based on the monomers 1.4-butanediol, adipic acid and terephthalic acid. It will biodegrade to the basic monomers 1,4-butanediol, adipic acid and terephthalic acid and eventually to carbon dioxide, water and biomass when metabolized in the soil or compost under standard conditions [50]. Specifically, PBAT C1200 has a density of 1.26 g/cm^3^ and MW = 126,000 g/mol. PBSA (BioPBS FD92PM) was supplied as pellet from Mitsubishi Chemical Corporation. It consists of a copolymer of succinic acid, adipic acid and 1,4-butandiol [51]. In particular, BioPBS FD92PM has a density of 1.24 g/cm^3^.

Treated calcium carbonate (further indicated as CC) (OmyaSmartfill 55—OM) was supplied from Omya SPA and is characterized by 55% of particles with a diameter less than 2 μm.

Wheat native starch was supplied by Sacchetto SPA (Lagnasco, CN, Italy). Poly(ethylene glycol) with Mn = 400 (PEG400) and Glycerol were purchased from Sigma-Aldrich (Milan, Italy). Absolute ethanol was purchased from Bio Optica S.p.A. (Milan, Italy). Immortalized human keratinocytes (HaCaT cells) were purchased from Cell Lines Service GmbH (Eppelheim, Germany). Resazurin sodium salt (used to prepare AlamarBlue test reagent), phosphate-buffered saline (PBS), fungizone and trypsin were provided by Sigma-Aldrich (Milan, Italy). Dulbecco’s modified Eagle’s medium (D-MEM), l-glutamine, penicillin–streptomycin (penstrep) and fetal calf serum (FCS) were obtained from Invitrogen (Carlsbad, CA, USA). Tri Reagent was purchased by Sigma-Aldrich/Merck (Darmstadt, Germany). LC Fast Start DNA Master SYBR Green kit was provided by Roche Applied Science (Euroclone S.p.A., Pero, Italy).

### 2.2. Preparation of Samples and Films

Preparation of PHA/starch films was accomplished in the following steps [14]: (1) Starch was mixed with glycerol and PEG 400 in an oven at 80 °C for 16 h to obtain its pregelatinization; (2) PHA powder was added to the starch in order to obtain a homogenous powder; (3) films were prepared through compression molding for 1 min at 190 °C.

Preplasticization of starch was performed by mixing wheat starch (RH 75%), PEG 400 and glycerol in a 60:10:30 ratio into a mortar. The mixture was kept overnight in a ventilated oven at 80 °C to obtain the starch gelatinization. Preplasticized starch (P-PLS) was then mixed with the powder of PHA in a 50:50 ratio into a mortar. Samples E-BM1, E-BM2, E-BM3, E-BM4 and E-BM5 were obtained by extrusion of the powder composed by PHA, pregelatinized native wheat starch (P-PLST), PBAT or PBSA and CC in a Minilab II Haake TMRheomex CTW 5 conical twin-screw extruder (Haake, Vreden, Germany). Materials were mixed for 1 min at the temperature of 140 °C and with a screw speed of 60 rpm. Torque values were recorded during all the extrusion process. The composition of the different blends is reported in Table 1. Some strands obtained from the extrusion were recovered and characterized.

The extruded strands (approximately 4 g for each extrusion) were minced and transferred between two Teflon square sheets for film preparation. The mixture was placed into the compression molding equipment at 190 °C, applying no pressure for the first 30 s, followed by the application of 4 metric tons for 30 s. After that pressure was removed, each film was rapidly removed and quenched with a cold air flow. Formed films were finally detached from the Teflon sheets. Several films were prepared for successive tests. Some nonextruded film samples were also prepared. They were produced only by compression molding the reference formulation named BM.

### 2.3. Characterization

#### 2.3.1. Material Characterization

Melt flow index (MFI) value was measured with a CEAST Melt Flow Tester M20 (Instron, Canton, MA, USA) equipped with an encoder. Samples were conditioned for 3 h in an oven at 60 °C before the tests. MFI is defined as the weight of molten polymer passed in 10 min through a capillary of known diameter and length, applying pressure through a weight. Measure was performed according to the ISO 1133:2005 and, in particular, the ISO1133D. In this work, a weight of 2160 kg was used, and melt volume rate (MVR) data were recorded each 6 s for 60 s. Different measurements of MVR were performed, and a mean value (with their standard deviation) was reported. MFR was calculated starting from MVR value, and its standard deviation was estimated by MVR standard deviation data multiplied for the viscosity in the melt (ratio of MFR to MVR).

From the starting films, some small square specimens of about 20 mm side length were cut in two replicates for each formulation. The squares were poured at room temperature in distilled water for 30 min. After soaking with water, the specimens were dried in an oven at 60 °C until constant weight. The replicates were left in water for 16 h and then washed and dried in the same way.

Before and after release in water, the surface morphology of films was analyzed by stereomicroscopy using a Wild Heerbrugg M3 microscope equipped with a Pulnix TMC-6 camera (Heerbrugg, Switzerland) and by scanning electron microscopy using an FEI Quanta 450 FEG scanning electron microscope (SEM) (Thermo Fisher Scientific, Waltham, MA, USA). Samples for SEM analysis were cryo-fractured and covered with a tiny metallic layer of Au, in a way that the surface could be electrically conductive, to observe the sectional surface.

Furthermore, before and after tests of release in water, films were also characterized by infrared spectroscopy using a Nicolet T380 Thermo Scientific instrument equipped with a Smart ITX ATR accessory with diamond plate (Thermo Fisher Scientific, Waltham, MA, USA). The spectra were normalized in intensity with respect to the band at 1720 cm^−1^ typical of PHA polymer.

#### 2.3.2. Epidermal Cell Culture and Viability Assay

HaCaT cells were cultured in D-MEM supplemented with 1% penstrep, 1% glutamine and 10% fetal calf serum at 37 °C in air and 5% CO_2_. The HaCaT cells, seeded in 12-well plates until 80% of confluence, were incubated for 24 h with the film (BM or E-BM5). At the end of this time, resazurin solution (AB) diluted in DMEM to a final concentration of 0.5 mg/mL was added to the cells and incubated for 4 h in an incubator. Resazurin incorporates a redox indicator that changes color according to cell metabolic activity. The supernatants were read with a spectrophotometer using a double wavelength reading at 570 and 600 nm. Finally, the reduced percentage of the dye (%AB_RED_) was calculated by correlating the absorbance values and the molar extinction coefficients of the dye at the selected wavelengths, following the protocol provided by the manufacturer.

#### 2.3.3. Evaluation of Immunomodulatory Properties

The immunomodulatory properties of BM and E-BM5 films were analyzed using HaCaT cells.

The cells, cultured as described above, were seeded inside 12-well TC plates until 80% confluence and were incubated for 24 h with the films for 6 and 24 h (*n* = 3). At these endpoints, total RNA was isolated with TRizol and 1 µm of RNA was reverse-transcribed into complementary DNA (cDNA) using random hexamer primers, at 42 °C for 45 min, according to the manufacturer’s instructions. Real-time polymerase chain reaction (PCR) was carried out with the LC Fast Start DNA Master SYBR Green kit using 2 µL of cDNA, corresponding to 10 ng of total RNA in a 20 µL final volume, 3 mM MgCl2 and 0.5 µM sense and antisense primers (Table 2). Real-time PCR was used to evaluate the expression of interleukins IL-1α, IL 1β, IL-6 and IL-8, as well as the expression of TNF-α and TGF-β.

## 3. Results

**Figure 1:**
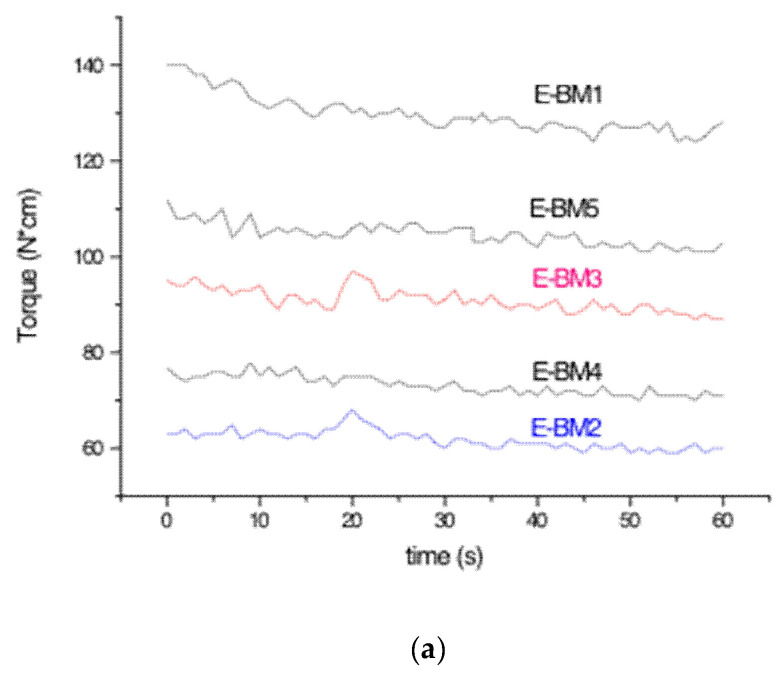

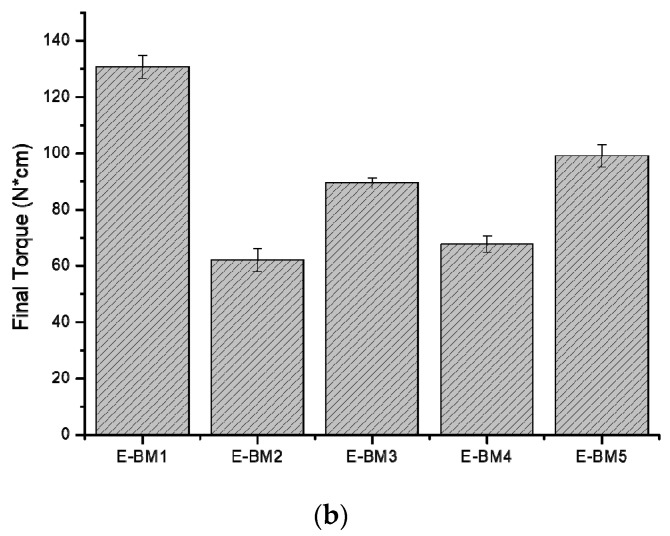
(**a**) Torque trends as function of extrusion time of the trial reported in Table 1; (**b**) final torque values of different extruded formulations.

In order to have more information about the possibility of producing beauty masks from films obtained by flat die extrusion, which is considered an affordable process for the scaling up of biobased film production, it was necessary to establish if the formulation containing P-PLST/PHA 1/1 with CC could also be extruded. The Minilab was set at 140 °C and 60 rpm, and the torque was recorded during the 1 min extrusion. The torque values were recorded along the extrusion trial (Figure 1a), showing a good melt stability of the prepared blends despite a slightly decreasing trend attributable to some chain scission of the processed polyesters [52]. The ribbon-like extruded strands recovered after extrusion were controlled, and their elasticity was qualitatively evaluated by deforming them using tweezers. The E-BM1 could be extruded, as reported elsewhere [14]. However, the study of its extrusion evidenced that it was not elastic and had a very high torque (Figure 1). Therefore, for this formulation, the scaling up to flat die extrusion is not possible.

A set of blends containing PBSA or PBAT replacing the PHA fully or at 50% was prepared, as reported in Table 2. All the blends contained 46.5 wt% of preplasticized starch (P-PLST). The replacement of the PHA with PBSA (or PBAT) led to a more elastic melt, with a lower torque, which seemed more suitable for flat die extrusion. The final torque values are reported in Figure 1b, where it is evident that both PBSA and PBAT reduced the torque in the same way.

The extruded blends were thus characterized to evaluate the possibility of scaling up the extrusion by melt flow rate test at 160 °C and weight of 2.16 kg. The obtained results are reported in Table 3. It is possible to observe that the pure PHA did not flow at all at 160 °C. When plasticized starch and calcium carbonate are added (E-BM1), the materials can flow at 160 °C, but the MFR is quite low (close to zero), in good agreement with torque data. The viscosity is thus very high, and this composition is thus not suitable for a possible scaling up in flat die extrusion equipment.

When PHA was replaced with PBSA, the viscosity decreased and the MFR increased at the value of 8.9 g/10 min. The values observed for E-BM2 and E-BM4 indicated that the strategy of using these PBSA or PBAT polymers as additives of PHA was valid, as these polymers show a significantly lower melt viscosity than PHA (E-BM1) in the presence of starch. In fact, if only half of PHA was replaced by PBSA (E-BM3 blend) the MFR was 2.3 g/10 min. The use of PBAT to fully or partially replace the PHA led to a similar trend. Therefore, the strategy of partially replacing PHA with PBSA or PBAT seemed to successfully modulate melt viscosity.

**Figure 2:**
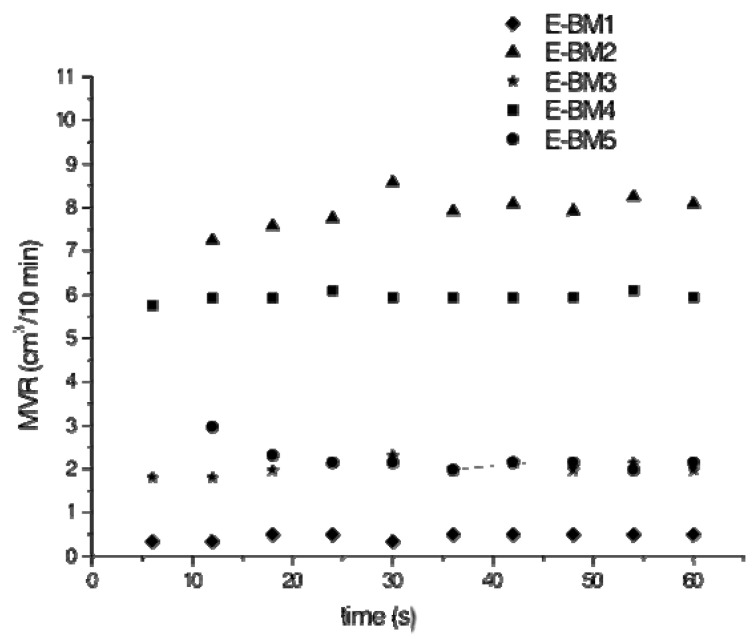
MVR as a function of time, recorded during the MFR tests.

The MVR values of the blends were stable as a function of time, as reported in Figure 2. Consequentially, the successive melting and flowing of the blends at 160 °C did not result in a significant chain scission of the biopolyesters, in agreement with torque trends (Figure 1a).

These data can be fundamental for scaling up the flat die extrusion in a semi-industrial plant. A processing temperature range between 140 and 160 °C can be suitable for E-BM3 and E-BM5, obtained by replacing half of the PHA with PBSA and PBAT, respectively, showing an improved processability with respect to the P-PLST/PHA/CC (E-BM1) blend.

**Figure 3:**
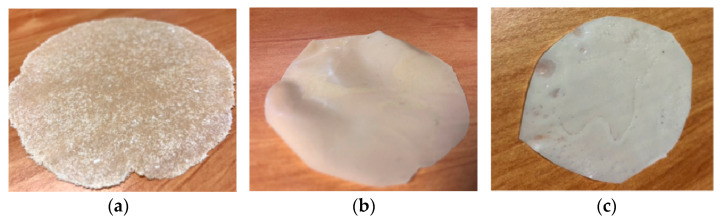
Compression-molded films: (**a**) BM without extrusion; (**b**) E-BM1; (**c**) E-BM5.

Films were obtained by compression molding and were extremely homogeneous and semi-transparent with respect to BM, obtained without extrusion (Figure 3). The composition of BM is the same as E-BM 1. The highest homogeneity was reasonably due to the better mixing achieved in the melt polymer, with the elongation flow generated by screws.

At least two replicated square specimens (30 mm side length) of each sample were immersed for 10 s in water and positioned on the forehead of a volunteer in a vertical position, noting the time that the wet pad remained on the subject’ forehead. Average resistances for E-BM1, E-BM2, E-BM3, E-BM4 and E-BM5 were 65, 7, 15, 3 and 60 min, respectively. E-BM5 displayed a similar behavior regarding adhesion to skin as BM1 but had an improved processability thanks to the partial replacement of PHA with PBAT. The blends E-BM1 and E-BM5 can be used for making beauty masks that are easy not only to wear but also to remove.

On the whole, taking into account the requirements of the final application, E-BM1 and E-BM5 seemed the best formulations for an easy-to-wear beauty mask, even though E-BM1 cannot be processed easily by flat die extrusion. So, the alternative BM (compression-molded) remains the best alternative.

The release of starch in water was studied for E-BM1 and E-BM5 and compared with that of BM to understand if the extrusion and the different compositions influenced the capability of starch to impart a rapid release to the films (Table 4). Interestingly, it was found that more than 80% of the extractable mass loss was lost in the first 30 min. This result regarding the short release kinetic is positive for the final application as a beauty mask. A slight reduction in the mass release was observed comparing BM and E-BM1, despite their identical composition. The extrusion, which allowed to obtain a more homogeneous sample thanks to a better dispersion of starch domains in the biopolyester matrix, reasonably made it less available to be released in water. However, the released mass amount is quite relevant, suggesting that the water extraction is still efficient in E-BM1. The presence of PBAT only slightly reduced the mass loss at 30 min, attributable to a slight decrease of release velocity that is due to the good affinity observed between PBAT and starch [53].

**Figure 4:**
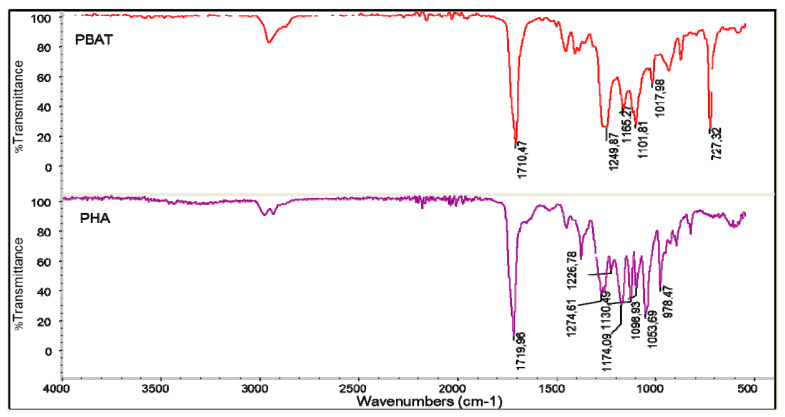
Infrared spectra of PBAT (red) and PHA (purple).

In Figure 4, the infrared spectra of PBAT and PHA are reported. The stretching C=O peak of PBAT has a maximum intensity at 1710 cm^−1^, whereas that of PHA is at 1720 cm^−1^. Moreover, the peak at 727 cm^−1^, typical of bending vibration of CH-plane of the benzene ring [54], was strong in intensity in PBAT and absent in PHA.

**Figure 5:**
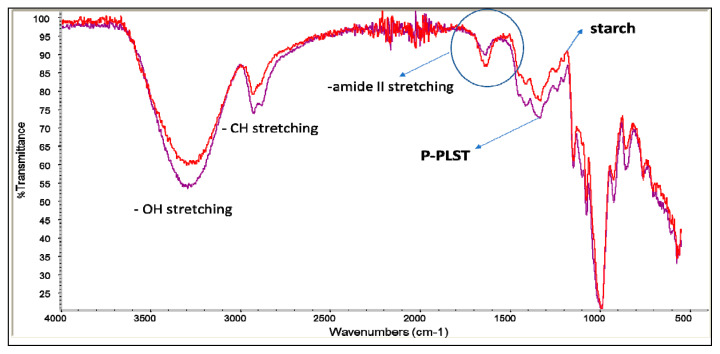
Infrared spectra of starch (red) and plasticized starch (purple).

In Figure 5, the spectra of native wheat starch and pregelatinized native wheat starch (P-PLST) are reported. The infrared spectra are quite similar because the macromolecular primary structure is not modified because of the pregelatinization that occurred. The increase of intensity of some bands is due to the addition of glycerol and poly(ethylene glycol) (PEG) to the native starch. In particular, the increase of intensity of the band at 3300 cm^−1^ (O–H stretching) is attributable to the increase of concentration of -OH groups due to the addition of both glycerol and PEG. A similar interpretation for the increase of the band at 2900 cm^−1^, due to C-H stretching, can be considered. The intensity of the peak at 1000 cm^−1^, attributable to C-O stretching, was not significantly modified because of the addition of glycerol and PEG, in good agreement with their molecular structure. Interestingly, the peak at 1640 cm^−1^, attributable to protein content of native starch [55], was decreased thanks to pregelatinization, essentially because of dilution due to the addition of glycerol and PEG.

**Figure 6:**
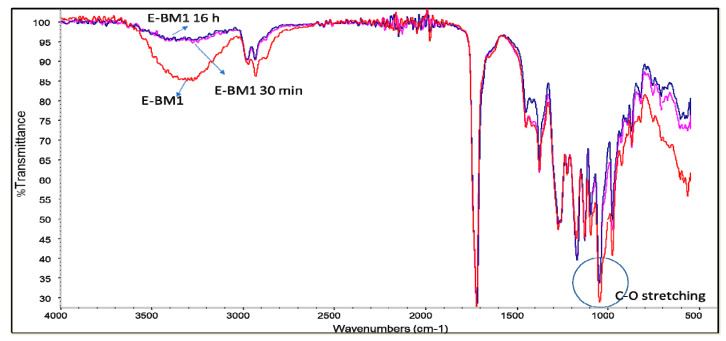
Infrared spectra of E-BM1 (red), E-BM1 immersed in water for 30 min (pink) and E-BM1 immersed in water for 16 h (blue).

In Figure 6, the spectra of E-BM1 before and after the immersion tests of 30 min and 16 h are reported. The reduction of the typical peaks of starch at 3350 and 1000 cm^−1^ (Figure 5) in the spectra of the films immersed in water can be easily noticed. The similar profiles of the spectra of E-BM1 after 30 min and 16 h are in agreement with the extraction data (Table 3). Interestingly, the bands typical of PHA (C-H stretching at 2900 cm^−1^) (Figure 4, PHA spectrum) are more evident in these spectra, as the release of starch from the film surface increased the concentration of biopolyester in the sample.

**Figure 7:**
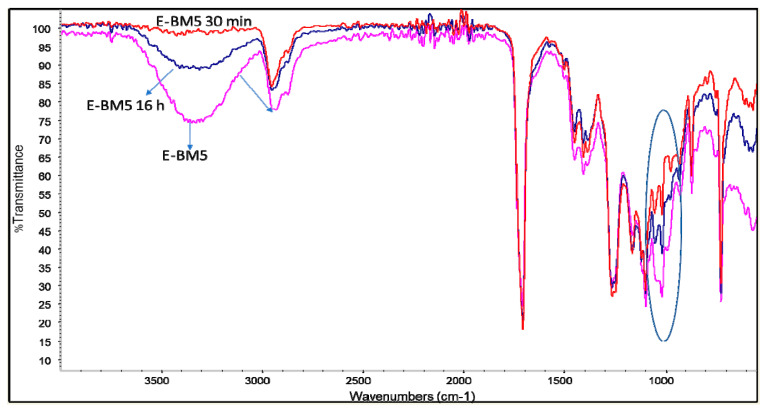
Infrared spectra of E-BM5 (pink), E-BM5 immersed in water for 30 min (red) and E-BM1 immersed in water for 16 h (blue).

A similar conclusion can also be drawn for E-BM5, where only the bands typical of starch decreased due to immersion in water, whereas the bands typical of PHA and PBAT were not modified. Interestingly, in the 1000 cm^−1^ spectrum region, after the release of starch, the two peaks at 1054 and 1102 cm^−1^ of PHA and PBAT, respectively, (Figure 7, circle) appeared quite evident.

**Figure 8:**
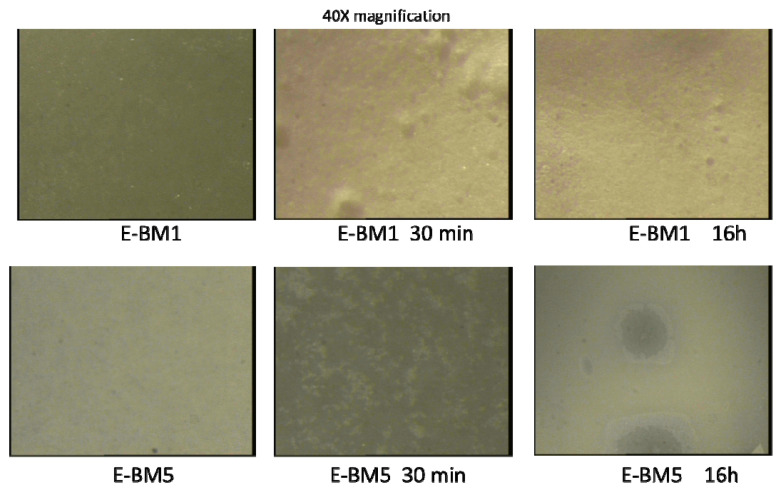
Analysis of E-BM1 and E-BM5 carried out by stereomicroscopy (40× magnification).

Regarding the analysis of the surface of E-BM1 and E-BM5 films by stereomicroscopy, the surface appeared smooth and homogeneous before the treatment in water. After the treatment for 30 min, some small holes and surface roughness changes can be noticed, and after 16 h the film remained similar (Figure 8). Therefore, as noticed by visually observing the improved homogeneity, the extrusion has made the blend of starch with the other polymers (PHB and PBAT) more homogeneous by dispersing it more finely in the polymeric melt.

**Figure 9:**
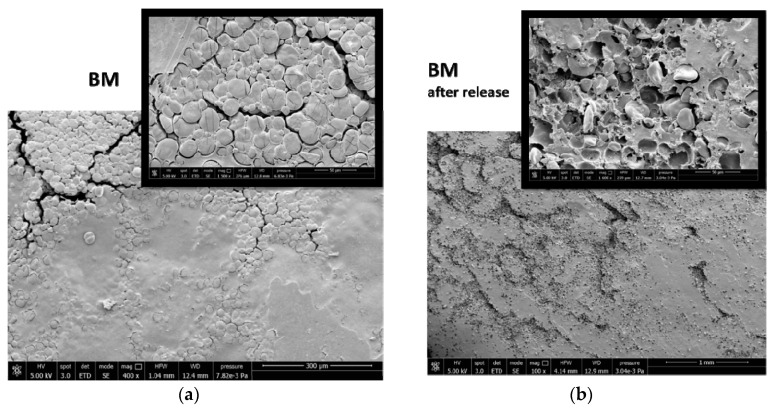
SEM micrographs of the BM film surface: (**a**) before the release in water at magnification 400× and 1500×; (**b**) after 16 h of release in water at magnification 100× and 1600×.

**Figure 10:**
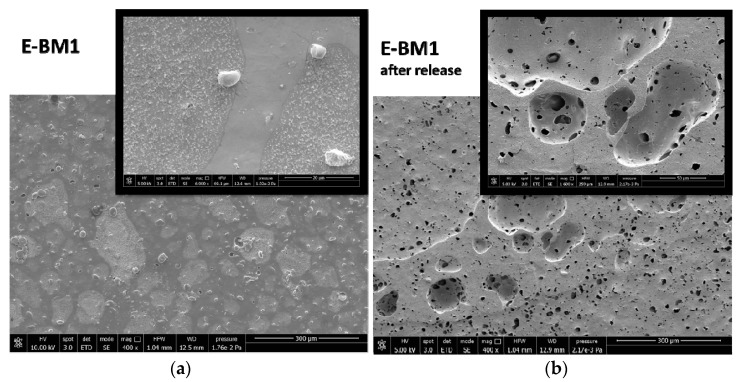
SEM micrographs of the BM film surface: (**a**) before the release in water at magnification 400× and 6000×; (**b**) after 16 h of release in water at magnification 400× and 1600×.

**Figure 11:**
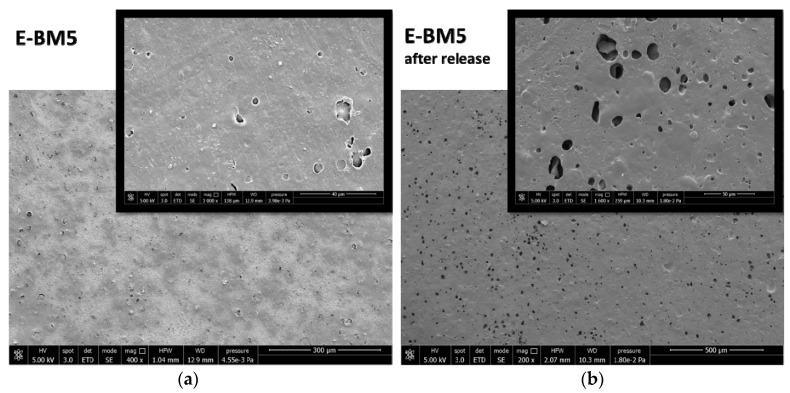
SEM micrographs of the BM film surface: (**a**) before the release in water at magnification 400× and 3000×; (**b**) after 16 h of release in water at magnification 200× and 1600×.

The surface properties of BM were different from those of E-BM1 and E-BM5, as the samples obtained without extrusion were less homogenous [16], as evidenced in Figure 3. To better understand the differences in between the films’ release behavior, the morphology of the E-BM1 and E-BM5 films was compared with that of BM using scanning electron microscopy (SEM) (Figure 9, Figure 10 and Figure 11).

Regarding BM obtained by compression molding the powder consisting of P-PLST, PHA and CC, a very rough morphology can be observed, where the round granules of starch were still present as such. The short processing by compression molding did not allow the full plasticization of the starch. In good agreement in the sample analyzed after the release in water, the presence of round holes evidenced, in agreement with infrared results, the release of almost spherical starch domains. In Figure 9b, at low magnification, it is evident that the starch granules are distributed in clusters. This is reasonably the cause of the inhomogeneity typical of this film (Figure 3a). On the other hand, the fast and efficient release is explained by the low adhesion between the starch granules and the biopolyester matrix.

The morphological structure of E-BM1 showed a reduced number of starch granules in good agreement with a very good dispersion and efficient plasticization of starch achieved thanks to the extrusion. The starch was mainly present as big domains of more than 100 µm. A good adhesion between the starch domains and the PHA matrix can be observed (Figure 10a, 6000× magnification). After the extraction, it can be confirmed that the starch domains are very big, as big holes of more than 100 µm can be revealed on the surface. Reasonably, the high viscosity of the blend does not allow a very fine dispersion of the plasticized starch domains into the PHA matrix. On the contrary, a more homogenous morphology in which micrometric starch domains are dispersed in the biopolyester matrix can be observed in the case of E-BM5 film. These domains are certainly more difficult to be extracted from the film and suspended in water because they are finely dispersed in the material bulk, and this can explain the slightly reduced release velocity of E-BM5.

As both PHA and PBAT are insoluble in water, the micrographs (Figure 11b) show the surface of the films consisting of the two polyesters. The two polyesters cannot be easily distinguished, and this can indicate a good compatibility between the two polyesters. However, the conditions to observe the phase morphology in a biphasic blend include the treatment through a cryogenic (brittle) fracture, better evidencing the interfaces. In this investigation, only the morphology of the films surface was considered, as it is specifically related to the beauty mask efficiency (release onto skin).

Regarding the compatibility with skin, the best films (compression-molded BM and E-BM5) sustain a high metabolic activity of keratinocytes (Table 5).

**Figure 12:**
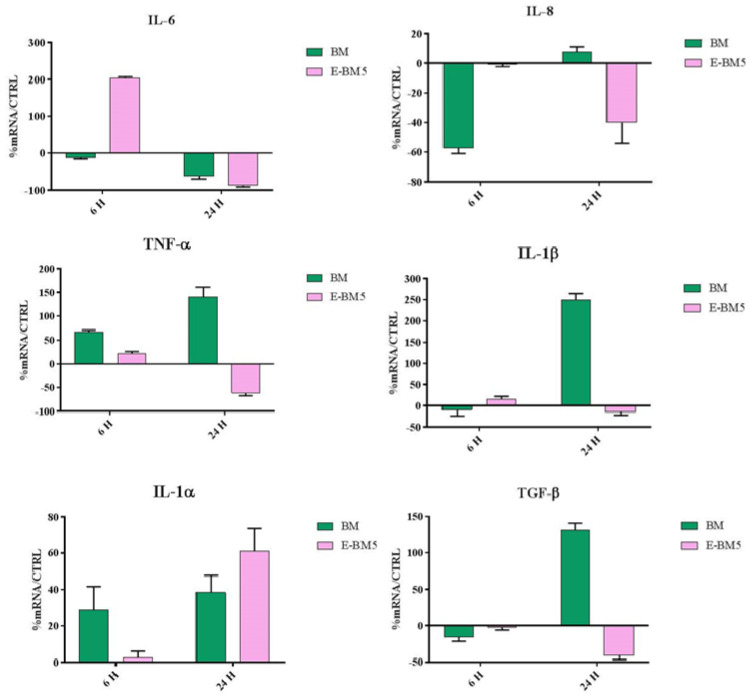
Relative gene expression of TNF-α, IL-8, IL-6, IL-1β, IL-1α and TGF-β in HaCaT cells incubated with BM and E-BM5 for 6 and 24 h. Data are mean ± SD and are expressed as percentage of increment relative to untreated HaCaT cells (control).

Moreover, these two films possess a significant immunomodulatory activity; in fact, they are able to upregulate the expression of both pro- and anti-inflammatory cytokines (Figure 12). However, while BM seems to induce a very marked and protracted immunomodulation over time, E-BM5 induces a downregulation of the proinflammatory cytokines after 24 h, suggesting an initial wound healing activity, followed by a resolution of the skin damage with consequent reduction of the inflammatory state.

## 4. Discussion

As shown in Section 3, the use of PBSA or PBAT is advantageous to reduce the viscosity of the plasticized starch/PHA blends, thus expanding the processing methodologies applicable to these blends and favoring their use in large scale applications. However, an important difference can be underlined between PBSA and PBAT observing the values related to melt fluidity. In fact, the MVR of pure PBSA was 2.45 cm^3^/10 min but the MVR of E-BM2, where PHA was fully replaced by PBSA, was 7.9 cm^3^/10 min. This strong increase of MVR can be explained only considering that the PBSA is strongly affected by chain scission [56] with a reduction of the average molecular weight because of the presence of starch and its plasticizers [32] acting as nucleophiles towards the ester groups on the polyester backbone. The MVR of PBAT was 4.7 cm^3^/10 min, whereas E-BM4, where PHA was fully replaced by PBAT, showed an MVR value of about 5.94 cm^3^/10 min. The increase of MVR due to chain scission was thus reduced with respect to the one observed for PBSA. This can be attributed to the general lower tendency of the aromatic aliphatic copolymers to undergo chain scission during processing, as evidenced by Signori et al. [52]. Interestingly, when PHA was present in the blend, the issue of chain scission seemed completely overcome. On the other hand, PHA also undergoes chain scission in the presence of P-PLST, as evident considering the MVR of E-BM1 (0.54 cm^3^/10 min), which is higher than the MVR of pure PHA (0 cm^3^/10 min). Thus PHA, having a good compatibility with PBAT and PBSA because of chemical affinity, highly increases the melt viscosity of the binary blends despite of the chain scission due to the presence of P-PLST as third component. The possible modulation of melt viscosity by finely tuning PHA/biopolyesters blend compositions is thus a possibility suggested by the present results that could be exploited for specific applications in the packaging, cosmetic and biomedical fields and connected processing methodologies. The possibility of obtaining films by flat die extrusion could be relevant for producing beauty masks, cosmetic pads or personal care films based on bioplastics [17]. Flat die extrusion can be better in the case that the production of beauty masks should be done considering a bigger scale than the one considered in compression molding. Flat die extrusion, consisting in the production of a film by a continuous methodology, is very fast. In this way, about 20 m of films is easily produced in one minute. Consequentially, the time necessary for producing a mask is more due to cutting and packaging than film production. In the case of compression molding, a maximum amount of 38 thousand pieces/year can be estimated, whereas the production of masks by an automatized flat die extrusion plant can produce more than 600 thousand pieces/year. This makes the process more competitive than the current industrial methodologies to produce films or nonwoven tissues for beauty masks.

The adhesivity to skin of the different films after the immersion in water is completely different. E-BM1 showed the maximum adhesivity to skin, and good performances were observed only in the blends where PHA was present. These results evidence the necessity of using PHA to grant a good skin adhesivity of the film. This is probably linked to the very high flexibility of this elastomer, which has a very irregular primary structure including hydroxybutyrate and hydroxyvalerate repeating units in similar atomic percentage [57]. The other biopolyesters showed a reduced capacity of adhering to the skin. Interestingly, the combination of PBAT and PHA resulted in good performances. PBSA and PBAT resulting as less skin-adhesive with respect to PHA has never been observed and explained before. On the other hand, the process of adhering to skin, slightly drying and successive detaching of the film is complex. The skin biochemical affinity, the flexibility of the films linked to their structure and viscoelastic behavior and their surface area can be considered as some important factors influencing this effect.

**Figure 13:**
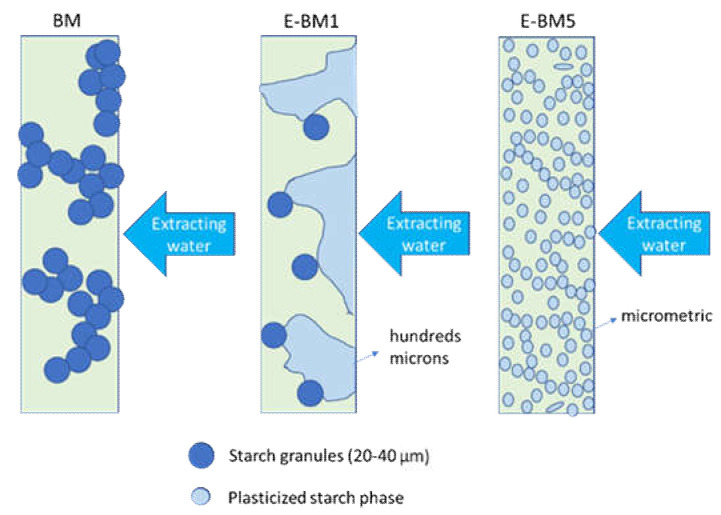
Scheme regarding morphologies of films linked to their release mechanism.

Regarding the release, the most efficient film is the one obtained by compression molding (named BM). Its most inhomogeneous and rough nature and the analysis of morphology performed by SEM integrated with other results (infrared spectroscopy, stereomicroscopy) allowed to explain the different release mechanisms (Figure 13). BM released rapidly and efficiently thanks to high interconnections, despite its inhomogeneity, the E-BM1 released rapidly but less efficiently due to a decrease in interconnectivity of P-PLST domains and E-BM5 showed an efficiency similar to E-BM1 but with a slightly slower mechanism of release due to the lower dimension of the P-PLST domains. Nevertheless, in general, all the morphologies seemed quite efficient in releasing, thanks to the high P-PLST content, allowing the interconnection of different starch granules or domains.

E-BM1 is the blend with the highest melt viscosity. In a paper regarding extrusion of starch and biopolyesters [36], it is reported that the higher the mechanical energy during the extrusion process, the greater the likelihood of interactions between starch and biopolyesters, which may be related to the increased resistance and decreased hygroscopicity found for these films. Thus, by increasing the compatibility between starch and PBAT there is a reduction of starch–water interactions. This change in hygroscopicity can explain the differences observed between BM and EBM1 in the mechanism of release in water.

Immunomodulatory activity tests performed on human keratinocytes (HaCaT cells) have shown that BM and E-BM5 are able to modulate the expression of both pro- and anti-inflammatory cytokines.

This behavior can be explained by giving the films a role in the wound repair.

Wound healing is a complex process characterized by a series of overlapping events which, starting from an inflammatory state, through reepithelization and matrix formation, lead to a remodeling of the tissue.

Wound healing is composed of two main phases. In the first phase, the production of proinflammatory cytokines occurs. TNF-α and IL-1α represent the primary cytokines for proinflammatory responses, immediately released by keratinocytes upon wound healing.

TNF-α can induce the production of fibroblast growth factor FGF-7, suggesting that it can indirectly promote reepithelization [58,59], while IL-1 increases migration and proliferation of keratinocytes [60]. In addition, a direct effect of IL-1 release is the upregulation of IL-6, important in initiating the healing response, and/or IL-8 production, a chemokine which contributes to the regulation of reepithelization, tissue remodeling and angiogenesis [59].

The second phase is associated with growth-oriented cytokines and factors, including TGF-β. This anti-inflammatory cytokine helps initiate granulation tissue formation by increasing the expression of genes associated with ECM formation including fibronectin, the fibronectin receptor and collagenane protease inhibitors [61,62,63] by preventing hyperproliferation of keratinocytes after wound closure. It is also involved in up-regulating the angiogenic growth factor VEGF [64] and in collagen production (particularly type I and III) and is also a potent inhibitor of metalloproteinases MMP-1, MMP-3 and MMP-9 [61,63,65]. Interestingly, once the wound field is sterilized, TGF-β is able to deactivate superoxide production from macrophages in vitro [66]. This helps to protect the surrounding healthy tissue and prepares the wound for granulation tissue formation [67].

However, our results show that BM seems to have a much stronger immunomodulatory activity compared to E-BM5. This is probably ascribable to the rougher surface, with inhomogeneous concentration of starch, that is more suitable for cell adhesion and proliferation, but more studies should investigate eventual specific effects of PBAT in the E-BM5 formulation.

## 5. Conclusions

Blends of PHA, plasticized starch and biopolyesters (PBSA and PBAT) were investigated as possible films for preparing beauty masks. The addition of PBAT or PBSA resulted as a good strategy for an improved processability by extrusion of the blends at 140 °C. PHA/starch blends or ternary PBAT/PHA/starch blends resulted in a good adhesion to skin after wetting the film. The release of starch from the films was between 38% and 49% of the total amount, indicating that these films can release more than 80% of the released mass in 30 min, hence with a fast kinetic. Some observed differences in the release behavior were explained by considering the different morphologies, investigated by scanning electron microscopy, obtained by changing blends composition.

The biocompatibility and immunomodulatory behaviors of PHA/starch blends or ternary PBAT/PHA/starch blends showed that both films are not detrimental for keratinocyte viability. However, the compression-molded versions, more inhomogeneous in terms of surficial morphology, resulted in having a much stronger immunomodulatory activity compared to E-BM5.

The results were interesting for selecting materials suitable for obtaining beauty masks by adopting different processing methodologies. Further work should investigate the way to add functional additives suitable to be released onto skin to these films.

## Figures and Tables

**Table 1:** Extruded P-PLST/EM/PBAT or P-PLST/EM/PBSA blends prepared by micro-compounder.

Blends	P-PLST (wt%)	PHA (wt%)	PBSA (wt%)	PBAT (wt%)	CC (wt%)
E-BM1	46.5	46.5	-	-	7
E-BM2	46.5	-	46.5	-	7
E-BM3	46.5	23.25	23.25	-	7
E-BM4	46.5	-	-	46.5	7
E-BM5	46.5	23.25		23.25	7

**Table 2:** Real-time PCR conditions for HaCaT cells.

Gene	Primer Sequence	Conditions	Size (bp)
IL-1 α	5′-CATGTCAAATTTCACTGCTTCATCC-3′	5 s at 95 °C, 8 s at 55 °C,	421
	5′-GTCTCTGAATCAGAAATCCTTCTATC-3′	17 s at 72 °C for 45 cycles	
IL-1 β	5′-GCATCCAGCTACGAATCTCC-3′	5 s at 95 °C, 14 s at 58 °C,	708
	5′-CCACATTCAGCACAGGACTC-3′	28 s at 72 °C for 40 cycles	
TNF-α	5′-CAGAGGGAAGAGTTCCCCAG-3′	5 s at 95 °C, 6 s at 57 °C,	324
	5′-CCTTGGTCTGGTAGGAGACG-3′	13 s at 72 °C for 40 cycles	
IL-6	5′-ATGAACTCCTTCTCCACAAGCGC-3′	5 s at 95 °C, 13 s at 56 °C,	628
	5′-GAAGAGCCCTCAGGCTGGACTG-3′	25 s at 72 °C for 40 cycles	
IL-8	5-ATGACTTCCAAGCTGGCCGTG-3′	5 s at 94 °C, 6 s at 55 °C,	297
	5-TGAATTCTCAGCCCTCTTCAAAAACTTCTC-3′	12 s at 72 °C for 40 cycles	
TGF-β	5′-CCGACTACTACGCCAAGGAGGTCAC-3′	5 s at 94 °C, 9 s at 60 °C,	439
	5′-AGGCCGGTTCATGCCATGAATGGTG-3′	18 s at 72 °C for 40 cycles	

**Table 3:** Melt flow Rate (MFR) and melt volume rate (MVR) values for the different tested compositions.

Blends	MFR (g/10 min)	MVR (cm^3^/10 min)
PHA	0	0
PBSA	2.7 ± 0.1	2.45 ± 0.09
PBAT	5.1 ± 0.1	4.7 ± 0.1
E-BM1	0.53 ± 0.09	0.44 ± 0.08
E-BM2	8.9 ± 0.4	7.9 ± 0.4
E-BM3	2.3 ± 0.2	2.0 ± 0.2
E-BM4	7.2 ± 0.1	5.94 ± 0.09
E-BM5	2.3 ± 0.3	2.2 ± 0,3

**Table 4:** Mass loss test in water for BM, E-BM1 and E-BM5 formulations.

Blends	After 30 min (wt%)	After 16 h (wt%)	After 30 min with Respect to P-PLST (wt%)
BM	24.6	25.0	49.0
E-BM1	19.3	20.8	41.0
E-BM5	17.6	22.2	38.0

**Table 5:** Keratinocytes viability tests.

Sample	%AB_RED_
BM	105
EBM-5	98
